# Death from Antitoxin

**Published:** 1895-05

**Authors:** 


### DEATH FROM ANTITOXIN.

The *Medical Standard* has repeatedly pointed out that untoward effects are a fair gauge of efficiency. No remedy can be potent whose action does not try hidden defect in the vascular or other systems upon which its action is directed. To state that a remedy is without untoward effects, is equivalent to stating that it is without efficiency. In dealing with untoward effects, however, the constitutional effects of even a local disease must be excluded. Diphtheria is one of the diseases in which the attention of the physician has been too much directed to local symptoms, because of surgical bias of the period resultant on the blatant, specious, reflex hypothesis. The fact that cardiac paralysis was always imminent, that myocarditis occurred, that renal symptoms were present, that paralytic states followed diptheria, was known ever since Bretonneau gave diphtheria a place in nosology. These constitutional symptoms are now ascribed to the use of antitoxin by men who have hitherto, in defiance of all clinicians, regarded diphtheria as a local disease.

That antitoxin should have untoward effects, is inevitable from its potent constitutional effects. From the time of its introduction, these have been looked for and described. Their description was testimony alike to the conscientiousness of the observer and his clinical skill. A sudden anginaform death in Brooklyn, it is alleged, resulted from antitoxin. The patient was an adult woman, who was not confined to bed, but permitted to walk around the room. The antitoxin employed had been used in New York, Chicago, and Alton, Ill., without any but the skin untoward effects. The remainder of the specimen used in the alleged fatal case, when tested by impartial clinicians, was found destitute of any unusual effects. The question therefore arises, was this death due to antitoxin? No logical medical thinker would so claim. Fatal cardiac paralysis is always so imminent in diphtheria, that there is persistent warning against permitting patients to assume the erect posture; such paralysis is much more likely to occur in adults in whom constitutional symptoms are most serious. The claim that antitoxin has caused death in this instance, is decidedly unwarranted. The assaults on antitoxin made because of it, are clinically absurd, and medico-legally dangerous to the profession. Had Long's first patient died from shock during the first anæsthesia, where would have been anæsthesia? That antitoxin should be used with caution is, of course, a childishly trite therapeutic axiom.—*Medical Standard.*

